# The complete mitochondrial genome of *Lagocephalus sceleratus* (Tetraodontiformes; Tetraodontidae) and phylogenetic studies of Tetraodontidae

**DOI:** 10.1080/23802359.2018.1502633

**Published:** 2018-08-23

**Authors:** Hui Jiang, Bingjian Liu, Zhenming Lü, Liqin Liu, Li Gong

**Affiliations:** a National Engineering Laboratory of Marine Germplasm Resources Exploration and Utilization, Zhejiang Ocean University, No. 1, South Haida Road, Dinghai District;; b Zhoushan 316022, Zhejiang, China; 2 National Engineering Research Center of Marine Facilities Aquaculture, College of Marine Science and Technology, Zhejiang Ocean University, Zhoushan, People’s Republic of China.

**Keywords:** Lagocephalus sceleratus, silver-cheeked toadfish, mitogenome, phylogenetic relationship

## Abstract

The complete mitochondrial genome sequence of *Lagocephalus sceleratus* was determined. The complete mitochondrial genome was 16,444 bp in length and contained 13 protein-coding genes, 22 transfer RNA genes, two ribosomal RNA genes, and two non-coding region (the control region and the origin of light strand replication). The overall base composition was A 27.59%, C 31.43%, G 17.12%, T 23.85%. All protein-coding genes started with an ATG initiation codon, except COI used GTG. With the exception of *ND6* and eight tRNA genes, all other genes were encoded on the heavy strand. Additionally, the phylogenetic relationship of 37 Tetraodontidae species based on the complete genome was analyzed, and the result showed that *L. sceleratus* was clustered with other Lagocephalus species. These results would be useful for the investigation of phylogenetic relationship, taxonomic classification and phylogeography of the Tetraodontidae.

*Lagocephalus sceleratus* (Tetraodontiformes, Tetraodontidae) is widely distributed in the tropical Indian and Pacific Oceans (Okan Akyo 2005; Türker-Çakır *et al.* 2009). It is a Lessepsian invasive and poisonous fish species introduced into the Mediterranean Sea through the Suez Canal (Bentur *et al.* 2008; Bakopoulos *et al.* 2017). Furthermore, the tetrodotoxin (TTX) of *L. sceleratus* can be used in the pharmaceutical industry (Nader M. 2012). In order to better understand the classification and biological value of this species, we determined the complete mitochondrial genome (mitogenome) of *L. sceleratus* (GenBank accession number: MH550879) and constructed a phylogenetic tree of Tetraodontida. This study is expected to contributing to the classification, systematic evolution of *L. sceleratus* and further phylogenetic relationship of Tetraodontidae and Tetraodontiformes.

*L. sceleratus* was collected from the South China Sea (22°16'32”N 114°09'56”E) and stored in a refrigerator of −80 °C with accession number 20171015XG04. The specimen was identified based on the morphologic features and COI gene. Total genomic DNA was extracted from muscle tissue using the phenol-chloroform method (Barnett & Larson 2012) and then used for PCR amplification and sequencing. The phylogenetic analysis of 37 Tetraodontidae was conducted based on the neighbor-joining (NJ) method using MEGA5 with 10,000 bootstrap replication (Tamura *et al.* 2011).

The complete mitogenome sequence of *L. sceleratus* was 16,444 bp in length, consisting of 13 protein-coding genes, 22 transfer RNA (tRNA) genes, two ribosomal RNA genes (12S rRNA and 16S rRNA) and two non-coding region (the control region and the origin of light strand replication). Except *ND6* and eight tRNAs (Gln, Ala, Asn. Cys, Tyr, Ser, Glu, Pro), other genes were encoded on the heavy strand. The overall base composition was A 27.59%, C 31.43%, G 17.12%, T 23.85%. The gene arrangement was consistent with other Tetraodontidae mitochondrial genomes (Yue *et al.* 2009; Jiang *et al.* 2014; Liu *et al.* 2014; Gong *et al.* 2016; Li *et al.* 2016). Twelve protein-coding genes started with an ATG initiation codon, *COI* used GTG as an initiation codon. For the termination codon, eight protein-coding genes (*ND1, ND2, ATP8, ATP6, COIII, ND4L, ND5, Cytb*) ended with TAA, three protein-coding genes (*COI, COII, ND4*) with a single T, *ND3* with TAG and *ND6* with AGG. The 13 protein-coding genes were 11,397 bp in length, accounting for 69.31% of the complete mitogenome, which encodes 3,799 amino acids in total. The 12S rRNA was located between tRNA^Phe^ and tRNA^Val^ and the 16S rRNA was located between tRNA^Val^ and tRNA^Leu^. They were 948 bp and 1,673 bp in length, respectively. The control region, located between tRNA^pro^ and tRNA^Phe^, is 955 bp. The origin of light strand replication was located between tRNA^Asn^ and tRNA^Cys^ with 51 bp in length.

The phylogenetic relationship of *L. sceleratus* and other 36 Tetraodontidae species was constructed using the neighbor-joining (NJ) method based the complete mitogenome. The result showed that *L. sceleratus* first clustered with other two Lagocephalus species, *Lagocephalus laevigatus* and *Lagocephalus spadiceus*, suggesting *L. sceleratus* had a closer relationship with *Lagocephalus* species (Figure 1).

**Figure 1.**
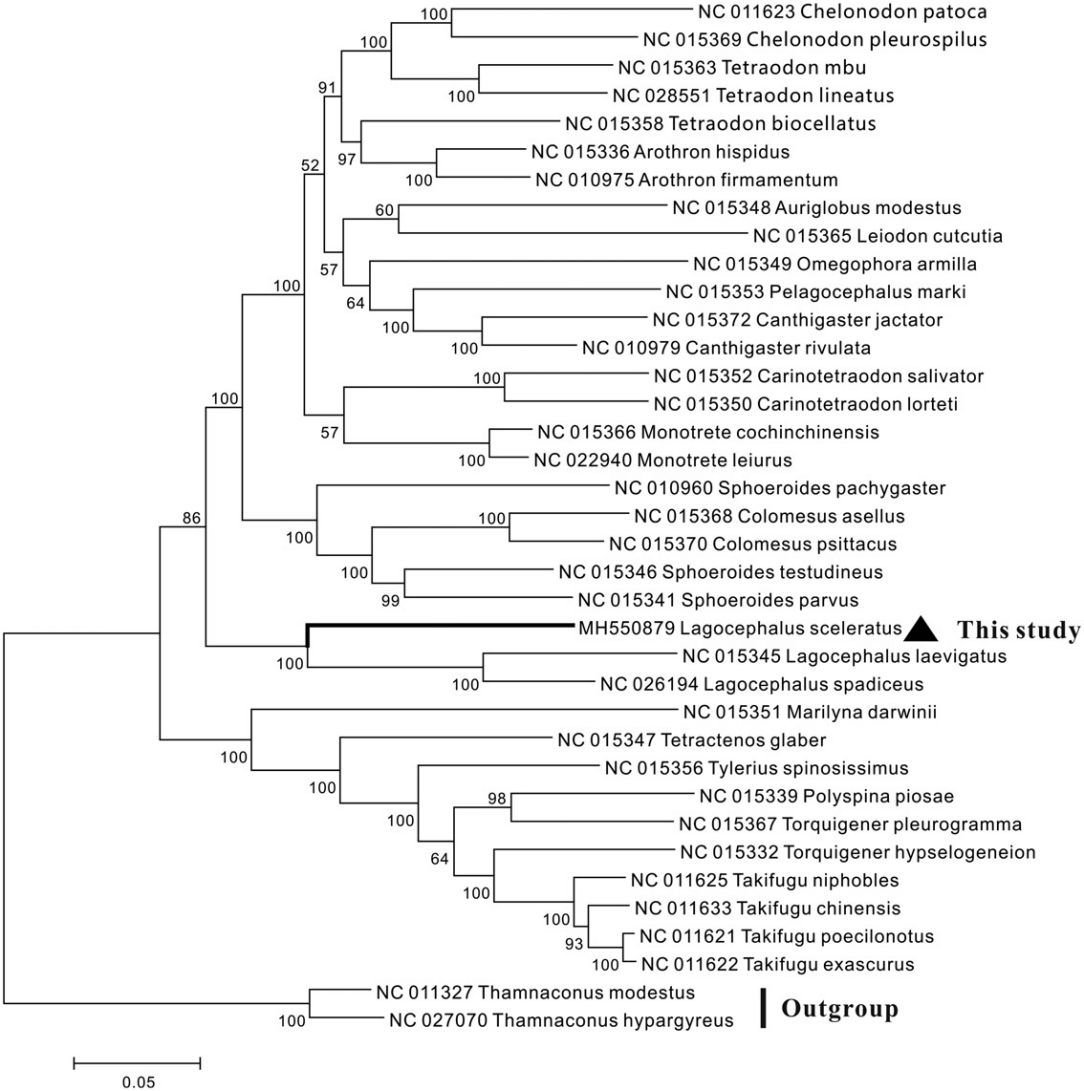
Neighbour-joining tree constructed based on the complete mitogenome of 37 Tetraodontidae species. The number at each node is the bootstrap probability. The number before the species name is the GenBank accession number
